# Prenatal cocaine exposure and its influence on pediatric epigenetic clocks and epigenetic scores in humans

**DOI:** 10.1038/s41598-024-52433-5

**Published:** 2024-01-23

**Authors:** Thiago Wendt Viola, Christina Danzer, Victor Mardini, Claudia Szobot, João Henrique Chrusciel, Laura Stertz, Joy M. Schmitz, Consuelo Walss-Bass, Gabriel R. Fries, Rodrigo Grassi-Oliveira

**Affiliations:** 1 School of Medicine, Developmental Cognitive Neuroscience Lab, Pontifical Catholic University of Rio Grande Do Sul (PUCRS), Porto Alegre, RS Brazil; 2 Translational Neuropsychiatry Unit, Department of Clinical Medicine, Aarhus University, Palle Juul-Jensens Boulevard 11, A701-129, 8200 Aarhus, Denmark; 3 Clinical Hospital of Porto Alegre, Porto Alegre, RS Brazil; 4 Faillace Department of Psychiatry and Behavioral Sciences, Translational Psychiatry Program, The University of Texas Health Science Center at Houston, Houston, USA

**Keywords:** Epigenomics, Risk factors

## Abstract

The investigation of the effects of prenatal cocaine exposure (PCE) on offspring has been inconsistent, with few studies investigating biological outcomes in humans. We profiled genome-wide DNA methylation (DNAm) of umbilical cord blood (UCB) from newborns with (n = 35) and without (n = 47) PCE. We used DNAm data to (1) assess pediatric epigenetic clocks at birth and (2) to estimate epigenetic scores (ES) for lifetime disorders. We generated gestational epigenetic age estimates (DNAmGA) based on Knight and Bohlin epigenetic clocks. We also investigated the association between DNAmGA and UCB serum brain-derived neurotrophic factor (BDNF) levels. Considering the large-scale DNAm data availability and existing evidence regarding PCE as a risk for health problems later in life, we generated ES for tobacco smoking, psychosis, autism, diabetes, and obesity. A gene ontology (GO) analysis on the CpGs included in the ES with group differences was performed. PCE was associated with lower DNAmGA in newborns, and this effect remained significant when controlling for potential confounders, such as blood cell type composition predicted by DNAm and obstetric data. DNAmGA was negatively correlated with BDNF levels in the serum of UCB. Higher tobacco smoking, psychosis, and diabetes ES were found in the PCE group. The GO analysis revealed GABAergic synapses as a potential pathway altered by PCE. Our findings of decelerated DNAmGA and ES for adverse phenotypes associated with PCE, suggest that the effects of gestational cocaine exposure on the epigenetic landscape of human newborns are detectable at birth.

## Introduction

In the last decades, the investigation of the long-term effects of prenatal cocaine exposure (PCE) on offspring development has been inconsistent with results showing small effect sizes, primarily when prenatal alcohol/tobacco exposure and socioeconomic factors are used as covariates^1^. The most consistent findings are related to infants being at risk for adverse perinatal outcomes^2^, motor development delays, and sudden infant death syndrome^3^. However, most of those studies have investigated cognitive or behavioral alterations of PCE through early childhood and adolescence, with very few investigating biological outcomes^1^.

On the other hand, rodent studies have shown that PCE is related to late-life phenotypes and epigenetic alterations^4^. In particular, PCE was associated with a shortened lifespan and other signs of altered development and age-related effects when rats were longitudinally evaluated. This included delayed earflap opening in postnatal life, a higher incidence of liver/spleen pathology later in life, and a shortened survival curve^5^. Despite a lack of investigations on the potential biological mechanisms to explain such findings, other studies found that PCE could induce profound structural and functional modifications in the epigenomic programs of neonatal and prepubertal mice^6^.

Unfortunately, the epigenetic effects of PCE in human populations are still unknown. Large-scale studies are challenging to perform because these populations are hard to find. The main issues are: (1) the biological samples should be collected before any potential postnatal factor could modify the epigenetic landscape, and (2) the PCE prevalence is estimated to be 0.15% in the general population when urine toxicology is used, and even lower when self-report methods are applied^7^. In contrast, DNA methylation (DNAm) profiling technology has advanced, allowing large-scale studies to investigate its role as a biomarker for several health-related phenotypes^8^.

To overcome these challenges, we profiled genome-wide DNAm of umbilical cord blood (UCB) from newborns with and without PCE in this study. To compensate for a relatively small sample size—but feasible to obtain in a single site—we used DNAm data to (1) assess their developmental maturity at birth and (2) build an individual’s risk for adult life disorders based on methylome data from previous large-scale studies. First, since DNAm at birth has proven to be an accurate predictor of gestational age—a proxy for assessing developmental maturity—we evaluated PCE effects on the gestational epigenetic age (DNAmGA)^9^. These age predictors use data from selected genomic cytosine-guanine dinucleotides (CpGs) sites, and their popularity has substantially grown because of their susceptibility to the effects of environmental exposures, clinical conditions, and health-related outcomes^10^. We also investigated the association between DNAmGA with UCB serum levels of brain-derived neurotrophic factor (BDNF), considering evidence suggesting that BDNF levels at newborn delivery could be linked to various clinical and maturational outcomes, including preterm birth^11^, fetal distress^12^, and neurodevelopment^13^. For instance, among preterm newborns, those with complicated postnatal development during the first year of life, including seizures, presented higher BDNF serum levels^14^.

Second, considering the large-scale DNAm data availability and existing evidence regarding PCE as a risk for health problems later in life, we generate epigenetic scores (ES) for tobacco smoking^15^, psychosis^16^, autism spectrum disorder (ASD)^17^, diabetes^18^, and obesity^19^ in adult life. We selected these phenotypes based on epidemiological evidence, which demonstrated the following associations: (a) Among those exposed to PCE, 30% developed tobacco use disorder by the age of 21^20^; (b) Cocaine use during pregnancy was linked to an earlier age at the onset of the first episode of psychosis^21^; (c) There was a higher incidence of autistic symptoms among babies exposed to PCE^22^; and (d) A positive association was found between PCE and the later development of type 2 diabetes^23^, accompanied by a four-fold increase in obesity^24^.

## Results

### Sample description

The demographic and clinical characteristics of the sample are presented in Table 1. The PCE group had lower newborn Apgar scores and birth weight than the control group. No group differences were found for newborn sex and gestational age (GA) assessment following birth. Mothers of the PCE group were older and had a higher frequency of non-white self-report ethnicity and infectious disease (HIV, hepatitis C, or syphilis) than control mothers. The frequency of tobacco smoking was significantly higher in the PCE group compared to the control group.

**Table 1:** Demographic and clinical characteristics of the sample.

Variables	PCE (n = 35)		Controls (n = 47)			
	Median/n	IQR/%	Median/n	IQR/%	Statistics	p-val
Newborn sex (male)	16	45.7	24	51.1	χ^2^ = 0.230	0.632
Gestational age at birth (weeks)	39	2.9	39.2	1.9	U = 976	0.193
Apgar	9	1	10	1	U = 1061	0.017
Newborn weight (grams)	2930	695	3018	600	U = 1039	0.044
Maternal infectious disease	16	45.7	0	0	χ^2^ = 26.69	0.001
Maternal ethnicity (white)	10	28.6	35	74.5	χ^2^ = 17.06	0.001
Maternal age (years)	26	6	24	7	U = 593	0.028
CD4T cell % estimate	0.132	0.083	0.001	0.014	U = 99	< 0.001
CD8T cell % estimate	0.134	0.078	0.067	0.037	U = 237	< 0.001
Neutrophils cell % estimate	0.516	0.182	0.841	0.080	U = 1547	< 0.001
Monocytes cell % estimate	0.090	0.031	0.078	0.028	U = 734	0.407
Nucleated red blood cell % estimate	0.460	0.110	0.560	0.050	U = 1426	< 0.001
ES for maternal smoking	0.59	1.36	− 0.44	1.08	U = 424	< 0.001
Frequent alcohol use (last trimester)	14	40.0%	–	–		
Frequent tobacco use (last trimester)	31	88.6%	4	8.5%	χ^2^ = 52.56	< 0.001
Frequent cannabis use (last trimester)	10	28.5%	–	–		
Frequent cocaine use (last trimester)	29	82.8%	–	–		

*PCE* Prenatal cocaine exposure, *ES* Epigenetic score, *IQR* Interquartile range, *χ*^*2*^ Chi-Square test, *U* Mann–Whitney *U* statistics.

Cell counts and percentages (CD4T, CD8T, Monocytes, Nucleated Red Blood Cells, and Neutrophils) were estimated based on DNAm data and compared between groups, showing striking differences in blood cell composition. Compared to control samples, PCE samples had more CD4T and CD8T cells. Lower Neutrophils and Nucleated Red Blood Cells were found in PCE samples compared to controls, while no group differences were found for Monocytes. In addition, as expected, the PCE group had a significantly higher ES for maternal smoking during pregnancy.

### PCE effects on epigenetic clocks

**Figure 1:**
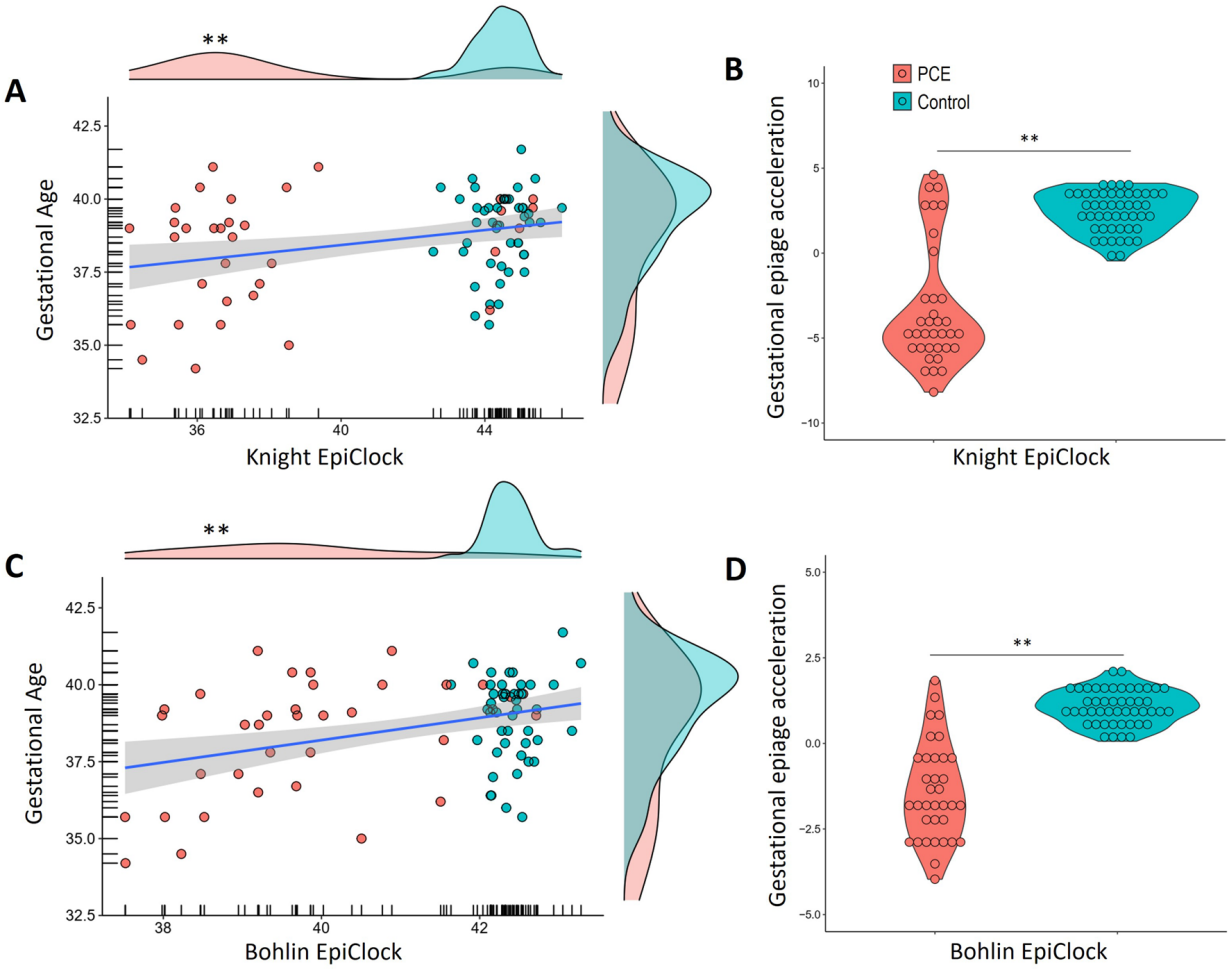
Prenatal Cocaine Exposure effects on epigenetic clocks. (**A**) Spearman’s correlation between gestational age and Knight epigenetic clock. (**B**) Group comparison in gestational age acceleration estimate using Knight clock (PCE median =  − 1.88; Interquartile range = 5.02/Control median = 5.71; Interquartile range = 2.91). (**C**) Spearman’s correlation between gestational age and Bohlin epigenetic clock. (**D**) Group comparison in gestational age acceleration estimate using Bohlin clock (PCE median = 0.68; Interquartile range = 3.10/Control median = 3.80; Interquartile range = 2.72). *GA* gestational age, *PCE* Prenatal cocaine exposure.

Cord blood DNAmGA was estimated based on Knight et al.^25^ and Bohlin et al.^26^ pediatric epigenetic clocks. DNAmGA was significantly associated with chronological GA using the Knight (rho = 0.28; p = 0.010; Fig. 1A) and Bohlin clocks (rho = 0.22; p = 0.043; Fig. 1C). Significant group differences were observed in both DNAmGA with PCE associated with lower ages in the Knight (U = 1416; p < 0.001) and Bohlin (U = 1540; p < 0.001) estimates. Epigenetic GA acceleration was estimated for both clocks, and a deceleration effect of PCE was detected, showing lower age acceleration in the Knight (U = 1423; p < 0.001; Fig. 1B) and Bohlin (U = 1533; p < 0.001; Fig. 1D) estimates in the PCE group compared to controls.

In generalized linear models with epigenetic GA acceleration as outcomes, we found that group status remained a significant predictor for both Knight and Bohlin estimates, even after adjusting for newborn Apgar score, newborn weight, maternal infectious disease, ethnicity, maternal age, ES for maternal smoking, and cord blood cell composition (CD4T, CD8T, nucleated red blood cells and neutrophils). Interestingly, we found that in addition to group status, ES for maternal smoking, CD8T, CD4T, neutrophil cell estimates, and maternal infectious disease and ethnicity were significantly associated with age acceleration in at least one analysis (Table 2). Both models outperformed the null models (Knight GLM model Omnibus χ^2^ = 125.2; p < 0.001/Bohlin GLM model Omnibus χ^2^ = 126.7; p < 0.001). We also performed two extra models, including sex as a covariate, and no sex or interaction effects were observed in either GA acceleration outcome.

**Table 2:** Generalized linear models predicting epigenetic gestational age acceleration.

GLM models	Knight epigenetic GA acceleration			Bohlin epigenetic GA acceleration		
*Covariates*	Beta	Wald	p-val	Beta	Wald	p-val
Apgar	− 0.20	1.40	0.237	0.118	2.08	0.149
Newborn weight	0.001	1.10	0.294	0.001	0.77	0.378
Maternal inf. disease	1.30	9.32	0.002	− 0.85	0.11	0.737
Maternal ethnicity	2.01	20.03	< 0.001	0.296	2.32	0.128
Maternal age	− 0.09	7.55	0.006	− 0.003	0.37	0.848
CD4T	− 11.4	3.99	0.046	− 1.152	0.14	0.701
CD8T	17.42	9.19	0.002	6.516	4.64	0.031
Neutrophils	9.45	18.3	< 0.001	5.643	21.85	< 0.001
nRBC	− 4.76	1.34	0.247	1.361	0.37	0.541
ES for maternal smoking	0.588	9.73	0.002	0.304	10.59	0.001
Group status	− 3.47	20.9	< 0.001	− 1.010	10.57	0.001

Group status about *PCE* Prenatal cocaine exposure, *ES* Epigenetic score, *CI* confidence interval, *Inf* infectious, *nRBC* nucleated red blood cells.

Sensitive analyses were performed regarding self-reported tobacco use. Participants who reported using tobacco during the last trimester of gestation demonstrated a significantly higher ES for gestational smoking (p < 0.001; U = 1271). As a result, we replicated the primary analyses using generalized linear models. However, in this replication, we replaced the ES for gestational smoking with self-reported tobacco use as a covariate. The findings were consistent, showing that the group status for PCE remained significant for both outcomes (Knight: Beta = 2.13; Wald = 4.24; p = 0.039/Bohlin: Beta = 0.93; Wald = 5.2; p = 0.021). Notably, in these analyses, self-reported tobacco use did not emerge as a significant predictor. Further sensitivity analyses were conducted, excluding the Apgar score and newborn weight from the analysis. This exclusion was justified by the lack of significant associations between these variables and outcomes in both Bohlin and Knight models, as indicated by the generalized linear models (Table 2). Despite removing these variables, all other covariates were retained. Similarly, the results demonstrated significant effects of the PCE group status on both the Bohlin (Beta = 0.90; Wald = 6.8; p = 0.009) and Knight (Beta = 1.91; Wald = 5.9; p = 0.015) models.

**Figure 2:**
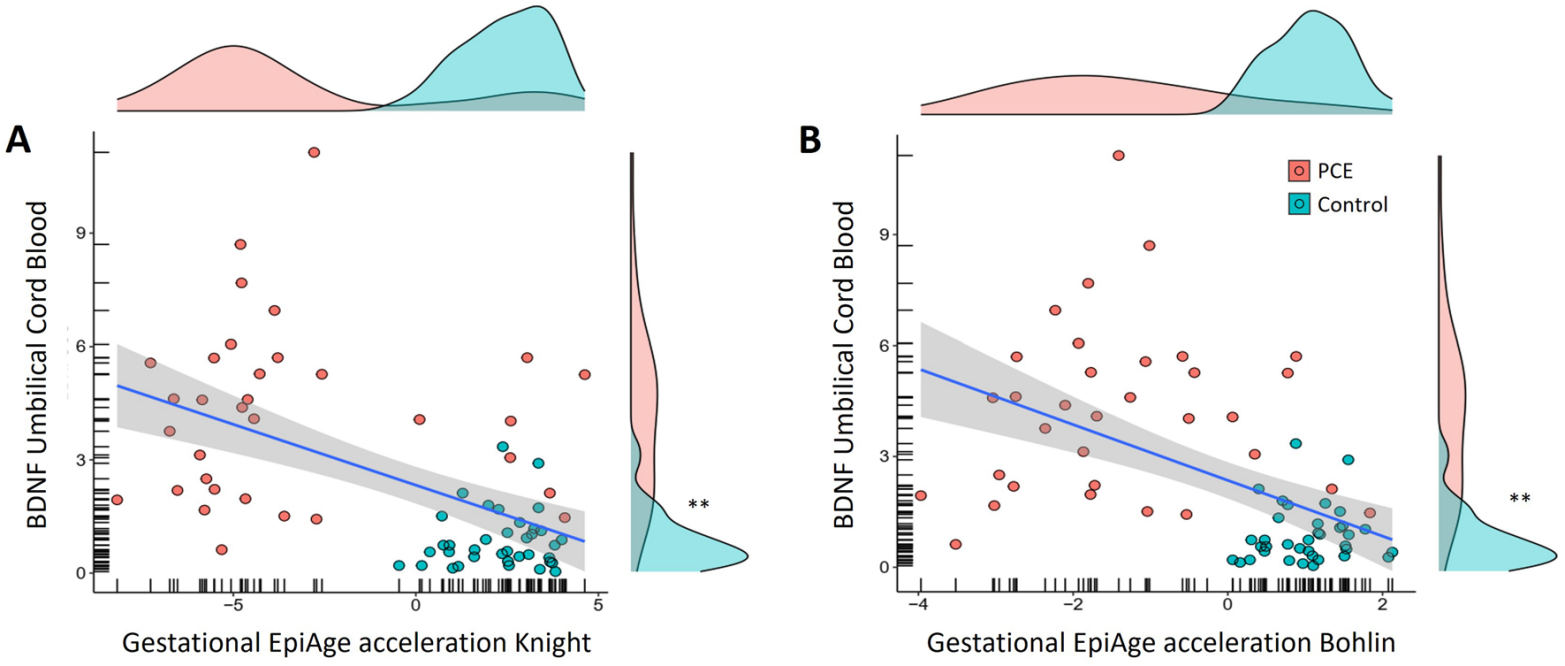
Correlation between epigenetic gestational age acceleration and BDNF. (**A**) Spearman’s correlation between BDNF levels in cord blood serum and Knight GA acceleration. (**B**) Spearman’s correlation between BDNF levels in cord blood serum and Bohlin GA acceleration. *GA* gestational age, *PCE* Prenatal cocaine exposure. **There are significant group differences in BDNF levels. Thirty-seven controls and 33 samples of newborns were exposed to PCE.

In addition, we tested whether epigenetic GA acceleration was correlated with BDNF levels in cord blood serum in a subsample (37 controls and 33 PCE samples). Higher BDNF levels in PCE samples were found compared to controls (U = 61; p < 0.001). Both Knight (rho =  − 0.49; p < 0.001; Fig. 2A) and Bohlin (rho =  − 0.55; p < 0.001; Fig. 2B) GA acceleration estimates were significantly negatively correlated with BDNF levels.

### PCE effects on epigenetic scores

**Figure 3:**
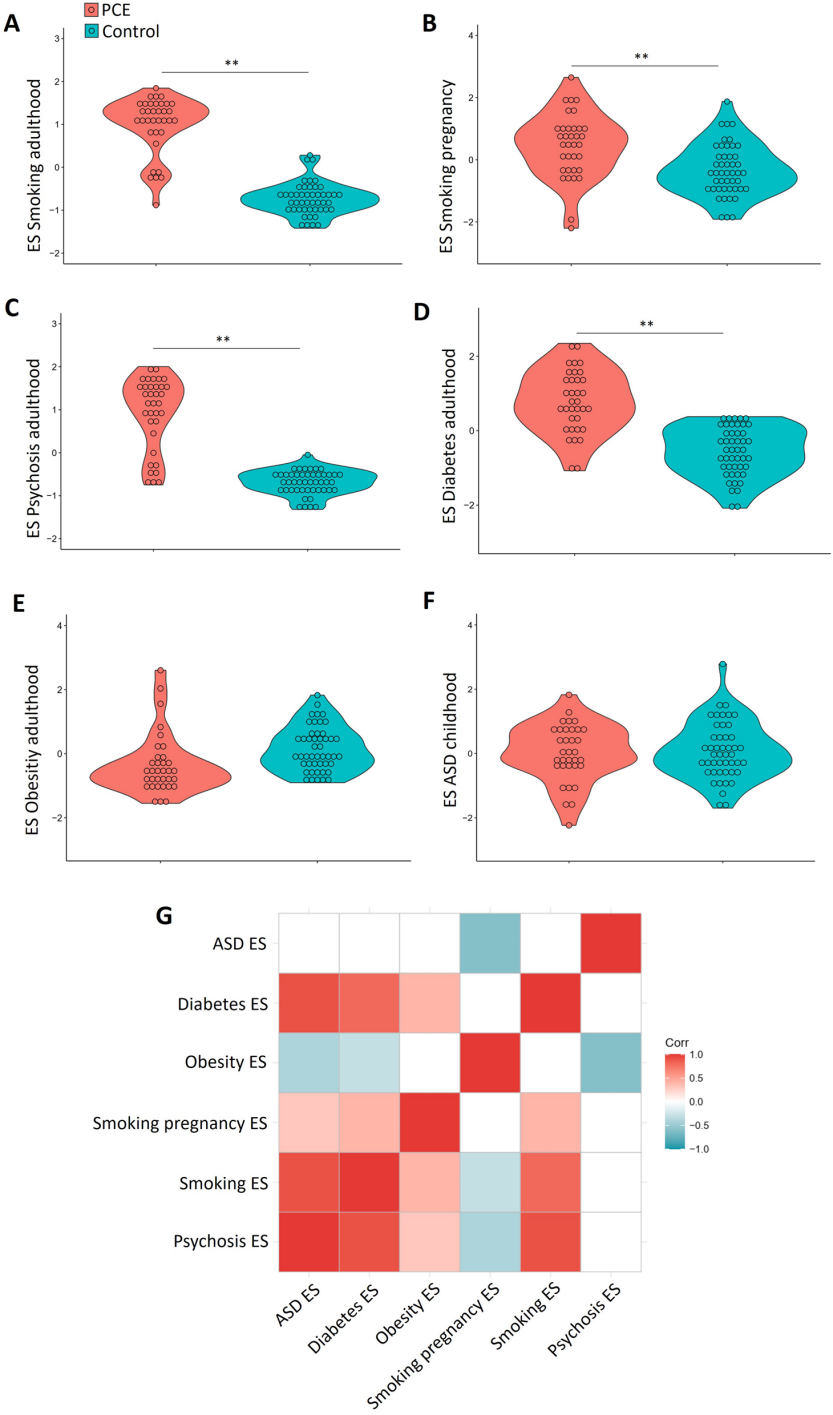
Prenatal Cocaine Exposure effects on epigenetic scores. (**A**) Epigenetic score for adulthood smoking. (**B**) Epigenetic score for gestational smoking. (**C**) Epigenetic score for adulthood psychosis. (**D**) Epigenetic score for adulthood diabetes. (**E**) Epigenetic score for adulthood obesity. (**F**) Epigenetic score for autism spectrum disorder during childhood. (**G**) In the correlation matrix between ES, insignificant correlations are depicted in white. *ASD* autism spectrum disorder, *ES* epigenetic score, *PCE* Prenatal cocaine exposure. **Significant group differences.

We found higher levels of ES in adulthood smoking (U = 43; p < 0.001), gestational smoking (U = 424; p < 0.001), psychosis (U = 140; p < 0.001), and diabetes (U = 83; p < 0.001) in newborns exposed to PCE (Fig. 3A–D). No group difference was found in the obesity score (Fig. 3E) and the ASD score (Fig. 3F). Correlation analyses showed significant positive associations between psychosis, adulthood/gestational smoking, and diabetes ES scores (Fig. 3G). Significant correlations were also found between ASD score, obesity score and psychosis score. Diabetes and obesity scores were also correlated. Details of group comparisons for each CpG probe, which were utilized to generate ES, including information about significant differences between groups and the genomic location of these probes, can be found in the Supplementary Material.

The GO analysis was performed with all CpGs used to generate ES of adulthood smoking, gestational smoking, psychosis, and diabetes by including the respective gene sets. No significant results were observed when analyzing the CpGs of each ES individually. When considering them collectively in a unified GO analysis, a significantly associated GO term was found: GABAergic synapses (Fold enrichment = 10.348; FDR = 0.092).

## Discussion

In this study, we investigated the effects of PCE on epigenetic measures of GA in UCB samples. We found that PCE was associated with lower epigenetic GA in newborns, and this effect remained significant when controlling for potential confounders, such as blood cell type composition predicted by DNAm and obstetric data. Epigenetic GA was negatively correlated with BDNF levels in the serum of UCB. In addition, we also investigated if PCE exposure could affect ES of smoking, psychosis, ASD, diabetes, and obesity, finding higher scores for smoking, psychosis, and diabetes. To our knowledge, this is the first study reporting epigenetic alterations due to PCE in humans.

Revealing factors that accelerate or delay epigenetic aging at birth may be useful to detect targets for disease prevention and improve newborn development throughout early life^27^. The Knight and Bohlin epigenetic clocks for GA prediction were developed to study UCB samples, and recent research has begun to reveal environmental and clinical factors associated with altered estimates of these clocks. Factors associated with deceleration in these epigenetic clocks at birth include maternal gestational depression, decreased C-reactive protein levels in the third trimester of pregnancy, vitamin D3 supplementation, and prenatal exposure to ambient air pollution^28^. PCE could decelerate epigenetic aging at birth due to its effects on reduced uterine blood flow with placental insufficiency, vascular disturbance, and decreased birth weight^29^, and these consequences could be associated with altered molecular mechanisms within the genome of the cell, such as DNAm^30^. In addition, preliminary findings of our laboratory in adult populations showed that cocaine use disorder was associated with accelerated epigenetic aging estimated by distinct clocks^31^, instead of a deceleration effect. Notably, there is a minimal intersection of probes among the three epigenetic clocks – Bohlin, Knight, and Horvath^32^. Specifically, the overlap is as follows: Bohlin to Horvath has 6 out of 148 probes (4%), Knight to Horvath has 1 out of 96 probes (1%), and Bohlin to Knight has 2 out of 96 probes (2%). While our data are suggestive, and additional studies are warranted, we could infer that the impact of chronic cocaine exposure on epigenetic clocks depends on the life stage. Despite that, a lower biological gestational age, in contrast to chronological gestational age, and a higher biological adult age, compared to chronological adult age, are both regarded as unfavorable states from a health perspective.

As mentioned earlier, both epigenetic clocks exhibit minimal overlap in CpG probes, indicating that they assess gestational age through distinct DNA methylation mechanisms. Notably, during their development, significant methodological differences were observed. The clock developed by Bohlin, with a training group five times larger in penalized regression models than the Knight clock, demonstrated superior performance in various cohorts^28^. This observation becomes particularly interesting considering our findings. In Bohlin's gestational age methylation data related to PCE, a deceleration effect was identified, with few additional significant effects of covariates (i.e., neutrophil cell estimates and the ES for gestational tobacco smoking).

On the other hand, the analysis of Knight's data also indicated a deceleration effect related to PCE analysis, revealing a broader range of significant covariates, including maternal infectious disease, ethnicity, age, cell estimates of CD4, CD8, and neutrophils, as well as the ES for gestational tobacco smoking. This suggests that the Knight clock is more sensitive to the influences of numerous potential factors. In contrast, the Bohlin clock appears responsive primarily to factors that substantially impact its age estimates.

Moreover, the deceleration effect associated with PCE was inversely correlated with circulating BDNF levels at birth, showing that PCE was associated with higher BDNF levels than non-exposed controls. Increased BDNF levels, in this case, might represent a compensatory mechanism for neurodevelopment and maturity^33^, or a resulting mechanism of chronic cocaine consumption. Indeed, in a case–control study comparing BDNF blood levels between women with and without cocaine use disorder, higher BDNF levels were found in women with the disorder^34^.

Although there are still uncertainties about whether DNAm might help to predict long-term risk for adverse health effects, risk scores based on DNAm data have been increasingly developed^35^. In our study, we generated ES for adulthood smoking, psychosis, diabetes, ASD, and obesity by using data from large-scale available studies of these conditions. PCE was associated with higher risk ES for smoking, psychosis, and diabetes. These ES also presented some degree of correlation. Epidemiological evidence supports that in-utero exposure to alcohol, tobacco, and illicit drugs could increase the risk of substance use disorder and psychotic symptoms later in life^36–38^. There is also evidence that the risk for the development of type 2 diabetes is increased by maternal substance use during the gestational period^23^. While the mechanisms underlying this effect are not well understood, we performed a GO analysis with the genes annotated to the CpGs used to estimate ES of adulthood smoking, gestational smoking, psychosis, and diabetes, to find gene pathways associated with this finding. While no significant results were observed when analyzing the CpGs of each ES individually, an interesting finding emerged when considering them collectively in a unified GO analysis. Altered GABAergic synapses were identified, implying that PCE might impact inhibitory synapses at some level. This is supported by animal studies showing that PCE could hinder the migration and maturation of GABAergic neurons^39,40^. Interestingly, the risk for smoking and polysubstance use is associated with altered GABAergic signaling during development^41,42^, as well as for schizophrenia^43^ and diabetes^44^. However, our findings of elevated risk for these conditions should be cautiously interpreted since ES may be more useful for contributing predictive value to multifactorial models in evaluating risk for disease development Field^8^ and not restricted to DNAm data per se.

This study has some limitations to be addressed. First, it has been previously shown that numerous factors, such as ethnicity and blood cell type composition, may be correlated with epigenetic age acceleration estimates using pediatric clocks^45^. In the generalized linear models, we observed that other variables, such as blood cell type composition and obstetric data, could influence the association with epigenetic age acceleration in addition to group status. Therefore, further studies with larger sample sizes could better investigate the individual contribution of each factor in addition to the PCE. Second, people with a history of cocaine use might also present a polysubstance use pattern, including chronic use of tobacco, alcohol, and other illicit drugs. We indeed observed that the ES for maternal smoking was associated with epigenetic age acceleration, and we cannot rule out the effects of the consumption of other substances in our findings. Third, we utilized adult epigenome-wide association studies (EWAS) data to estimate ES in newborns. There remains a lack of evidence regarding the long-term stability of these ES. Therefore, our findings should be interpreted cautiously, and future studies need to replicate our research using a larger sample. Longitudinal assessments of DNAm-based outcomes, especially about ES associations, are highly recommended. Fourth, while generating ES, we chose CpG probes with significant associations to phenotypes, setting the threshold at least as stringent as 1 × 10^–5^. It is important to note that using an arbitrarily chosen p-value threshold introduces some limitations in capturing the extent of variance in the disease/trait by the enrichment scores. Fifth, another limitation is the low correlation coefficients between epigenetic clock estimates and gestational age. Most previous reports used ultrasound data for estimating gestational age. In our study, we could only employ Capurro’s method. This approach estimates gestational age by evaluating the physical characteristics of the newborn, such as skin texture, amount of lanugo (fine hair on the body), breast development, and the formation of the genitalia, within the first few hours of a newborn's life, but it may not be as accurate as ultrasound. Sixth, we lacked clinical data on mothers regarding outcomes such as psychosis and diabetes, for which ES significantly differed between groups. However, it is plausible that mothers suffering from e.g., psychosis tend to smoke more or overuse cocaine. Thus, future studies could address this limitation by including comprehensive data on these outcomes. Seventh, it should be noted that the associations found in UCB in our study may not be replicated in other sample sources due to the tissue-specificity of epigenetic mechanisms. Eighth, no data on the level of cocaine exposure was available, which hindered further analysis of potential correlations between this exposure and epigenetic outcomes. Ninth, there might be a common genetic component driving both an increased risk for PCE and ES for smoking, psychosis, and obesity, which are not investigated in our study. Also, current epigenetic age calculators do not consider the impact of common risk variants on epigenetic regulation.

## Conclusion

In conclusion, we found that PCE was associated with a delayed ticking of pediatric epigenetic clocks of GA, suggesting that the effects of in-utero exposure to cocaine on the epigenetic landscape of newborns are capable of being detected at birth. A higher risk for adverse health conditions, such as tobacco smoking, psychosis, and diabetes, was also found in risk scores developed based on DNAm data. Thus, our findings support the pre-clinical literature showing the consequences of PCE at the molecular level.

## Methods

### Sample

Umbilical cord blood from 35 newborns with PCE and 47 newborns without (Controls) were obtained from a biobank created to investigate the effects of gestational substance use^33,46^ in Southern Brazil. The study was performed by the principles stated in the Declaration of Helsinki. Before starting the study, ethical approval was obtained for all protocols from the local Institutional Review Board and Ethics Committee of the Pontifical Catholic University of Rio Grande do Sul. The study meets national and international human research guidelines. All participants agreed and signed an informed consent form.

Eligibility for the PCE group was to be a last-trimester pregnant woman between 18 and 45 years old with a history of cocaine use during gestation (snorted and/or smoked) and a positive urinary test (Bioeasy^®^ cocaine test, Alere™, BH, Brazil) confirming recent cocaine consumption. All mothers included in the control group self-reported being non-drug users and had negative toxicology urinary tests. Additionally, control mothers could not have a history of misuse of alcohol or other drugs, according to the Mini International Neuropsychiatric Interview (MINI)^47^. The Alcohol, Smoking and Substance Involvement Screening Test (ASSIST)^48^ was performed to inquire mothers about their behavior of consuming alcohol, tobacco, cannabis, or cocaine during pregnancy.

Sociodemographic perinatal and obstetric data were obtained through interviews or by review of medical records. The following maternal variables were used in this study: age, self-reported ethnicity, educational attainment, clinical status of relevant infections (syphilis, HIV, and hepatitis C), and substance use during pregnancy. Regarding newborns, the following variables were used: weight, Apgar score at 5 min, gestational age (GA) at neonatal examination assessed through Capurro’s method^49^, and assigned sex.

### DNA methylation

UCB was collected after the placenta was delivered by venipuncturing of the umbilical vein, according to the guidelines of the NetCord-FACT International Cord Blood Standards^50^. Blood samples were collected in anticoagulant-free tubes and centrifuged at 4.0 rotations per minute for 10 min at 4 °C for serum fraction separation. In contrast, the remaining lower fraction was stored for DNA isolation using the Gentra Puregene® Blood Kit (Qiagen, Hilden, Germany). Isolated DNA was quantified on NanoDrop (Thermo, Waltham, MA, USA), and 500 ng of DNA was bisulfite-converted using the EZ DNA Methylation Kit (Zymo Research, Irvine, CA, USA). Then, samples were evaluated using the Infinium Human Methylation EPIC BeadChip (Illumina, CA, USA) and scanned with an iScan Microarray Scanner (Illumina, CA, USA) according to their protocols.

We used the RnBeads R package^51^ to extract individual CpG site methylation data. Specifically, we ran standard quality control and preprocessing analysis, removing CpGs located in sex chromosomes, sites located in proximity to SNPs, and cross-reactive CpG sites. We also removed any probes that have failed in one or more samples and probes with p-values below 0.05 during the quality control, indicating a poor-quality signal. We used the minfi^52^ R package function preprocessQuantile for data normalization. Thus, 672309 probes were retained for analyses. The methylation percentage of individual CpGs is reported as a ß-value, a continuous variable between 0 and 1. To calculate epigenetic clocks, we used the Methylclock R package^53^. We generated DNAmGA and epigenetic GA acceleration through residuals obtained after regressing DNAmAge on GA for each sample based on Bohlin^26^ and Knight^25^ pediatric epigenetic clocks. Bohlin and Knight clocks were developed for predicting GA using cord blood samples. In addition, cord blood cell type composition (CD4T, CD8T, Monocytes, Nucleated Red Blood Cells and Neutrophils) was estimated using the EpiDish^54^ and minfi^52^ R packages.

The ES was generated using previous EWAS published data. Since more than 88% of PCE mothers were also frequent tobacco smokers during pregnancy, we constructed scores for maternal tobacco smoking using the same 19 probes reported in^15^. We then generated ES for (a) tobacco smoking during adulthood using the same 15 probes reported in Ref.^16^; (b) adulthood psychosis using the same 72 probes reported in Ref.^18^; (c) childhood autism spectrum using the same six probes reported in Ref.^17^; (d) adulthood diabetes using the same 68 probes reported in Ref.^19^; and (e) adulthood obesity using the same 41 probes reported in Ref.^55^. The selected probes were those that in the EWAS exhibited significant phenotype associations with thresholds set at least as stringent as 1 × 10^–5^, mirroring the thresholding approach commonly employed in polygenic risk scoring^56^.

We determined ES by calculating weighted values from CpG probes based on the published data. This process follows the methodology outlined in Ref.^57^. To assign weights to each CpG probe, we divided the CpG coefficient presented in the above papers by the average coefficient of all measured CpGs linked to the studied phenotype^35^. After estimating the weights, we included our data using the following equations. For CpG sites with increased methylation levels associated with the disease reported in the above papers, scores were calculated as follows: [(beta values in our sample—reference coefficient values) * weight]. Conversely, for CpG sites where decreased methylation levels were linked to the disease, scores were calculated as: [(reference coefficient values—beta values in our sample) * weight]. The ES was then obtained by summing up data from all CpG sites^58^ and undergoing Z-score transformation.

In addition, a gene ontology (GO) enrichment analysis was performed by including all individual CpGs used to estimate ES with significant group differences. The GO analysis was performed using the ShinyGO platform with the KEGG pathway database. GO terms with an FDR correction value of less than 0.1 were considered significant.

### BDNF levels

Umbilical cord serum was separated within two hours after blood collection by centrifugation at 4000 × *g* at 4 °C. BDNF levels were determined by sandwich-ELISA using monoclonal antibodies specific for BDNF (R&D Systems, Minneapolis, MN), according to the manufacturer's instructions^33^.

### Statistical analysis

The Wilcoxon-Mann–Whitney test or t-test assessed differences in continuous variables between groups, while differences in categorical variables were evaluated by Chi-Squared tests. Correlations were performed using Spearman’s rho coefficient. Given the non-normal distribution observed in a substantial portion of the variables, we employed Generalized Linear Models with an inverse Gaussian function and identity link function to predict epigenetic GA acceleration. This modeling approach included the incorporation of a group variable while controlling for covariates such as newborn Apgar score, newborn weight, maternal infectious disease status, ethnicity, maternal age, maternal smoking exposure, and cord blood cell composition (including CD4T, CD8T, monocytes, nucleated red blood cells, and neutrophils). A simple adjustment was made to the dependent variable values by adding 10 to them to facilitate the use of generalized linear models. This adjustment was applied to prevent negative values in the dependent variable during the modeling process. A p‐value < 0.05 was considered statistically significant. All analyses were performed using the open-source statistical software R (version 4.2.0), and graphs were developed using ggplot2^59^.

### Ethics approval and consent to participate

The study was performed by the principles stated in the Declaration of Helsinki. Before starting the study, ethical approval was obtained from the Institutional Review Board and Ethical Committee of the Pontifical Catholic University of Rio Grande do Sul. The study meets national and international human research guidelines. All participants agreed and signed an informed consent form.

### Supplementary Information

Supplementary Information.

## Data Availability

The datasets and the R scripts generated during the current study are available from the corresponding author on request.

## Abbreviations

PCE: Prenatal cocaine exposure
DNAm: DNA methylation
DNAmGA: Gestational epigenetic age
UCB: Umbilical cord blood
BDNF: Brain-derived neurotrophic factor
ES: Epigenetic scores
ASD: Autism spectrum disorder
GA: Gestational age
GO: Gene ontology
EWAS: Epigenome-wide association studies
